# Fungal Biomass Protein Production from* Trichoderma harzianum* Using Rice Polishing

**DOI:** 10.1155/2017/6232793

**Published:** 2017-03-06

**Authors:** Sibtain Ahmed, Ghulam Mustafa, Muhammad Arshad, Muhammad Ibrahim Rajoka

**Affiliations:** ^1^Department of Biochemistry, University of Agriculture, Faisalabad 38040, Pakistan; ^2^University of California San Diego, 9500 Gilman Drive, La Jolla, CA 92093, USA; ^3^Department of Bioinformatics and Biotechnology, Government College University, Faisalabad 38000, Pakistan; ^4^University of Veterinary and Animal Sciences, Sub-Campus Jhang, Lahore, Pakistan

## Abstract

Industrially important enzymes and microbial biomass proteins have been produced from fungi for more than 50 years. High levels of crude protein as much as 45% are present in fungal biomass with balanced essential amino acids. The aim of this study was to access the potential of* Trichoderma harzianum* to produce fungal biomass protein from rice polishings. Maximum biomass yield was obtained at 5% (w/v) rice polishings after 72 h of incubation at 28°C at pH 4. Carbon and nitrogen ratio of 20 : 1 gave significantly higher production of fungal biomass protein. The FBP in the 75 L fermenter contained 49.50% crude protein, 32.00% true protein, 19.45% crude fiber, 9.62% ash, 11.5% cellulose content, and 0.325% RNA content. The profile of amino acids of final FBP exhibited that all essential amino acids were present in great quantities. The FBP produced by this fungus has been shown to be of good nutritional value for supplementation to poultry. The results presented in this study have practical implications in that the fungus* T. harzianum* could be used successfully to produce fungal biomass protein using rice polishings.

## 1. Introduction

Bioconversion of the wastes is a natural way for the recovery of resources and the natural recycling process can be facilitated by biotechnology. Biotechnological treatments to food processing wastes which are found in large quantities can produce useful end products including microbial biomass protein (MBP) while the wastes are also purified during the process. In modern biotechnology industry the MBP production from raw materials is a field with the leading volume capacity and one of the most studied topics in the field of biotechnology [1]. In a sustainable society the carbohydrate by-product conversion into value-added products is very important for production from renewable energy resources. In Pakistan, cheap and nonconventional agricultural and industrial residues can be accumulated up to 50 million ton and fermented for production of single cell protein (SCP) [2, 3]. The use of microbial fermentation to cycle and recycle these residues will not only be resulted in the reduction of pollution but also help to produce low cost and high quality MBP. Production of MBP from wastes is helping to solve the world environmental and industrial waste problems [4]. Various agroindustrial wastes have been utilized for the production of MBP for poultry and livestock feeds. The products of MBP have been used for human and animal consumption as marketable products [5] and different fungi and yeast are known for the production of these MBP. Fungi have more advantages for MBP production as their pellet or filamentous morphology allows low cost to isolate and recover MBP from culture media that builds a substantial fraction of capital and operating costs to produce MBP. Various substrates can be used to grow fungi for MBP production. Zhang et al. [6] used starch wastes to produce MBP. However, in this study for the first time, we reported production of fungal biomass protein (FBP) using rice polishings.

Rice is one of the largest and important crops in the world and in Asia only, more than two million people obtain 60–70% of their calories from rice [7]. Rice polishing is a by-product of rice milling and is the most economical source for energy and protein in poultry feeding. Being a good source of protein, vitamins, minerals, and energy, rice polishing has great potential as an important ingredient in poultry feed [8]. It can be transferred into fermentable sugars through appropriate hydrolysis and can be utilized as cultural substrate to grow microorganisms. As a source of animal feed stock, the effective utilization of rice polishing will not only help to solve the problems of utilization of bioresources but also enable farmers to reveal new sources of income through selling value-added microbial biomass [7].

Due to abundance in nature the microorganisms are considered as natural producers of enzymes and are, therefore, important for biomass conversion [9]. Other than some bacterial species and yeast genera, filamentous fungi of* Aspergillus*,* Chaetomium*,* Paecilomyces*,* Penicillium*, and* Trichoderma* genera have also been used for biomass production [10].* Trichoderma harzianum* is a filamentous fungus and is important in biotechnological point of view [11]. For the production of maximum biomass it is necessary to optimize process parameters and to make it economically feasible. This study aims at producing fungal biomass protein from rice polishings using* T. harzianum*.

## 2. Materials and Methods

### 2.1. Chemicals and Substrate

All the chemicals used were of analytical grade unless otherwise stated. Rice polishings were used as a substrate and purchased from a local market. The substrate was defatted through repeated washings with petrol and then dried to a constant weight in an air oven at 60°C and packed in polyethylene bags for future use. The proximate analysis was performed to find out nutritive potential of defatted rice polishings using the method of Rajoka et al. [12].

### 2.2. Organism and Cultivation Conditions

The filamentous fungus* T. harzianum* was used in this study. The fungus was maintained on agar slants containing 0.2% malt extract, 0.2% yeast extract, 2% glucose, and 2% agar at 28°C. Freshly inoculated slants were incubated at 28°C for 5 days and stored at 4°C. Vogel's medium was used for inoculum preparation of* T. harzianum* [13]. Rice polishings were used as a substrate and inoculum of fungus was grown on an orbital shaker at 28°C (120 rpm for 24 h). 0.1% CaCl_2_·2H_2_O, 0.15% MgSO_4_·7H_2_O, and 0.2% KH_2_PO_4_ were also added in the medium for the optimal production FBP.

The ability of the organism to produce FBP from rice polishings as a sole carbon and energy sources was studied in optimized Vogel's medium in a 5 L fermenter (2.5 L working volume). The composition of optimized Vogel's medium was as follows: 5% (w/v) rice polishings, 5 g/L trisodium citrate, 2 g/L NH_4_NO_3_, 0.2 g/L KH_2_PO_4_, 0.2 g/L MgSO_4_, 4 g/L (NH_4_)_2_SO_4_, 1 g/L peptone, and 2 g/L yeast extract. The pH of all media was adjusted to 5.0 with 1 M NaOH or 1 M HCl [14]. Biomass samples were taken every day, steamed for 5 minutes, and filtered through coarse glass wool. The residues, thus obtained, were thoroughly washed thrice with distilled water and then dried at 60°C to a constant weight. The dried biomass was then ground to pass through 40-mesh sieve ad analyzed for crude protein content.

### 2.3. Optimization of Biomass Production from* T. harzianum*

Conventional process was applied for optimizing cultural condition by applying optimizing one parameter at a time approach as used extensively by several authors [5–8]. Different rice polishing concentrations were tested for to produce FBP from* T. harzianum*. The pH optima to produce FBP were checked at different pH values. Optimum temperature for FBP was also determined at different temperatures. Different ionic concentrations of CaCl_2_·2H_2_O, MgSO_4_·7H_2_O, and KH_2_PO_4_ were tested to get optimal FBP production through fermentation of rice polishings with* T. harzianum.*

### 2.4. Effect of Carbon Nitrogen Ratio on FBP Production

The concentration of soluble carbohydrate of growth medium was determined in terms of glucose that represents “C.” Total nitrogen of the growth medium was estimated through Kjeldahl's method. For constructing different C : N ratios, urea as nitrogen source and glucose as carbon source were adjusted accordingly. Different C : N ratios (i.e., 30 : 1, 25 : 1, 20 : 1, 15 : 1, 10 : 1, and 5 : 1) were examined to get the optimal C : N ratio for maximum FBP production from* T. harzianum.*

### 2.5. Production of Large Scale Biomass

The optimum conditions revealed for* T. harzianum* in 5 L fermenter were extended to ferment rice polishings in a 75 L fermenter (50 L working volume) to produce FBP and amino acids. The product of biomass obtained on large scale was dried in forced air oven at 60–65°C and examined.

### 2.6. Biological Evaluation of FBP

One-day-old broiler chicks (Hubbard) were purchased from the local market and raised on maize feed for ten days. Then thirty birds were selected on weight uniformity basis and were randomly allotted to rations A, B, C, and D in such a way that each ration was fed to six experimental birds. Each chick was kept in a separate pen in triplicate. The birds were individually wing tagged for identification. The initial body weight of the experimental birds ranged between 180 and 190 g. For biological evaluation, the FBP was fed to ten-day-old broiler chicks for a period of ten experimental days. To determine protein quality of biomass, it was replaced with soybean meal with the proportion of 30% and 60%, respectively.

For these studies above-mentioned four experimental rations, namely, A, B, C, and D were prepared. A control ration containing 23.0 percent crude protein and 3200 kcal metabolizable energy was designated as ration “A.” Rations B and C were formulated containing 23 percent crude protein and 3200 kcal metabolizable energy/kg. In ration B, 30 percent of the soybean meal was replaced with FBP while, in ration C, 60 percent of the soybean protein was replaced with FBP. A nitrogen free ration (D) was formulated having 3200 kcal metabolizable energy/kg. Initial body weight, feed offered and feed refused, and bird weight were taken into account to study feed conversation ratio, net protein utilization, and protein efficiency ratio as described earlier [15].

### 2.7. Analytical Methods

The FBP collected through fermentation of rice polishings by* T. harzianum* was tested following AOAC methods [16]. Aliquots (200 mL) were picked out at various time intervals for the determination of cell biomass protein, true and crude proteins. For this purpose, the whole content (200 mL) was homogenised and centrifuged (12,000 ×g for 10 min, 4°C) to remove substrate and cell mass. The substrate was separated from cell mass by low centrifugation (4,000 ×g for 15 min, 4°C). The cell mass formed upper layer and was separated from the substrate settled at the bottom. The substrate was washed thrice with distilled water and dried at 95°C to a constant mass. The cell mass was also washed thrice and suspended in saline and its quantity was determined from the measurement of protein as well as gravimetrically as described below.

One fraction of culture broth (100 mL) was also centrifuged at 10,000*g* for 10 min and saline solution was used to wash the cell pellet. The pellet was suspended in 10 mL dist. H_2_O and dried at 70°C to a constant mass. The other fraction was also centrifuged, washed twice with saline, and finally suspended in saline to determine its intracellular protein as described earlier [12].

### 2.8. Amino Acid Analysis

Standard method was used to determine amino acid composition. Briefly, the fermentation broth and fermentation product were dried at 70°C in a hot air oven. In a boiling water bath, one gram of sample was hydrolyzed with 1 N HCl (50 mL). The sample was then centrifuged at 10,000*g* to recover hydrolysate. This hydrolysate (100 *μ*L) was injected into amino acid analyzer (Evans Electro-Selenium Limited, UK) and quantified using the method of Rajoka et al. [17].

### 2.9. Determination of Kinetics and Thermodynamic Parameters

The kinetic parameters were studied as given by Aiba et al. [18]. The differential equations which captured the dynamics of mixed carbohydrates consumption (*S*), cell mass formation (*X*), and crude protein (CP) synthesis are given below:

(1) dXdt=μX,dCPdt=QCP×X,−dSdt=−μXYX/S−QCPXYCP/S,dPdS=YCP/S,dPdX=YCP/X,

where *Q*_CP_, *q*_*P*_, *Y*_*X*/*S*_, *Y*_CP/*S*_, and *Y*_CP/*X*_ are the volumetric rate of crude protein formation, specific rate of protein formation, substrate consumption yield coefficient with respect to growth, crude protein synthesis based on substrate utilization, and cell mass formation, respectively.

All the kinetics and thermodynamic parameters determined were also described briefly in our previous studies [17, 19].

### 2.10. Statistical Analysis

MStatC software was used to compare treatment effects through protected least difference method. Significance of difference was given in the form of least significant difference (LSD) values at *P* ≤ 0.05.

## 3. Results and Discussion

### 3.1. Optimum Conditions for FBP Production

With the view to obtain an increased FBP production, different fermentation conditions were optimized. These studies were made in a 5 L fermenter (2.5 L working volume) for the optimization of fermentation conditions to produce FBP from* T. harzianum*.

### 3.2. Effect of Rice Polishings on Biomass Production

**Figure 1:**
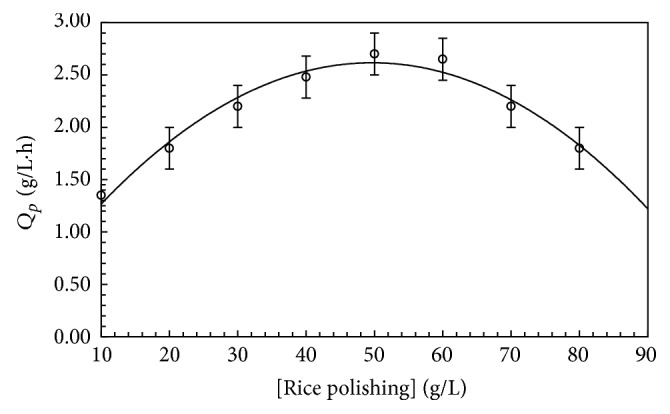
Effect of different rice polishings concentrations (% w/v) on FBP production by* T. harzianum* at pH 4 and 28°C after 72 h. Error bars show standard deviation among triplicate observations.

Among different rice polishings concentrations, 5% (w/v) rice polishings resulted in predominantly higher FBP production (Figure 1). FBP was not increased with further increase in concentration of substrate. Gradual reduction in formation of protein, substrate consumption, and cell mass synthesis rates were calculated when concentration of rice polishings was increased or decreased. Earlier it has been reported that 9% (w/v) rice polishings gave significantly higher FBP production [12]. Hence* T. harzianum* requires less amount of substrate for FBP production and is more economical in regard to industrial scale production.

### 3.3. pH and Temperature Effect on Biomass Production

**Figure 2:**
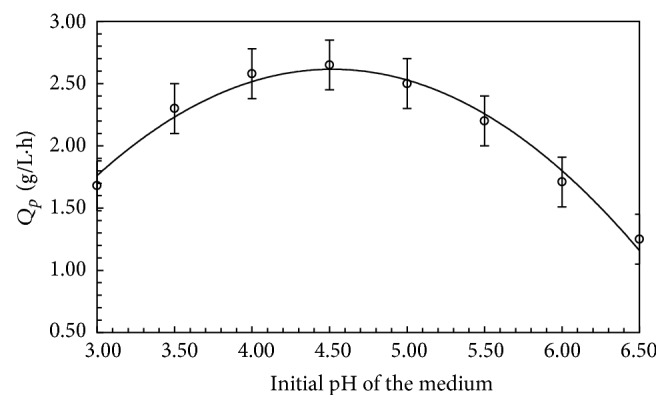
Effect of pH on FBP production by* T. harzianum.* Error bars show standard deviation among triplicate observations.

The production of FBP depends on the initial pH of the fermentation medium. The influence of pH on FBP production is shown in Figure 2. Maximum FBP production was obtained at pH 4 at 28°C. pH had a significant effect on the FBP production.* Candida utilis* gave maximum biomass production at pH 6.5 from wheat bran [19] and* Chaetomimum* sp. produced maximum protein at pH 5.5 [20]. The highest amount of FBP was produced by* T. viride* WEBL0702 at pH 4.5 [6]. A significant increase in the FBP yield from* Aspergillus niger* and* Rhizopus oryzae* was achieved when the initial pH was increased from 5.5 to 6 [10].

**Figure 3:**
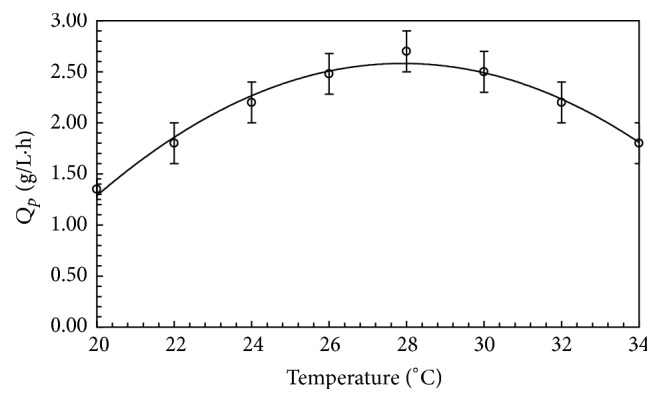
Effect of temperature on FBP production in 5 L fermenter under optimized fermentation conditions. Error bars show standard deviation among *n* = 3 readings.

Operating temperature is one of the most important factors in industrial applications to determine the production cost and product quality. The production of FBP by* T. harzianum* at different temperatures was checked. The effect of fermentation temperature on FBP production by* T. harzianum* is shown in Figure 3. Maximum FBP was obtained at 28°C. Further increase in temperature resulted in decreased FBP production. Earlier scientists have reported different results for the optimal fungal biomass protein production at different temperatures. The optimal growth temperature for the production of FBP was 27.5°C from* A. niger*,* R. oryzae,* and* Saccharomyces cerevisiae* [10].

### 3.4. Time Course of FBP Production from* T. harzianum*

**Figure 4:**
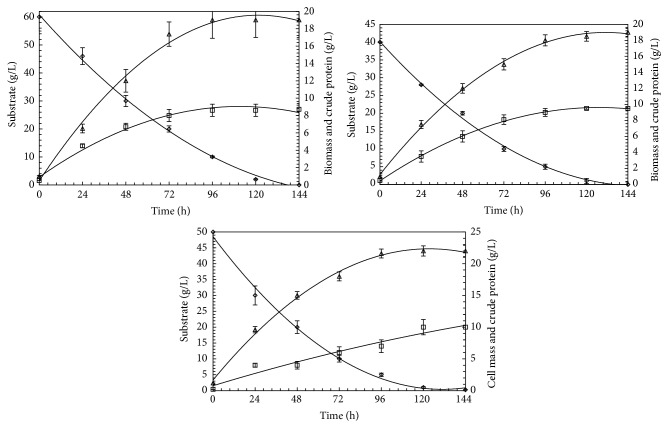
Time course of fungal crude protein and cell mass production by* T. harzianum* under preoptimized fermentation conditions. Error bars show standard deviation among *n* = 3 observations.

Time course for FBP production was investigated in Vogel's medium (initial pH, 4) with 5% (w/v) rice polishings as a substrate at 28°C. Representative time course of FBP production by* T. harzianum* from rice polishing is shown in Figure 4. Maximum FBP was obtained at 72 h of fermentation (Figure 4). The FBP reduced after its peak value if the fermentation was further carried out which could be because of cell autolysis. Hence optimum pH 4 and optimum temperature 28°C and optimum incubation time of 72 h were used in all the succeeding experiments.

### 3.5. Effect of Ionic Concentration on FBP Production

**Figure 5:**
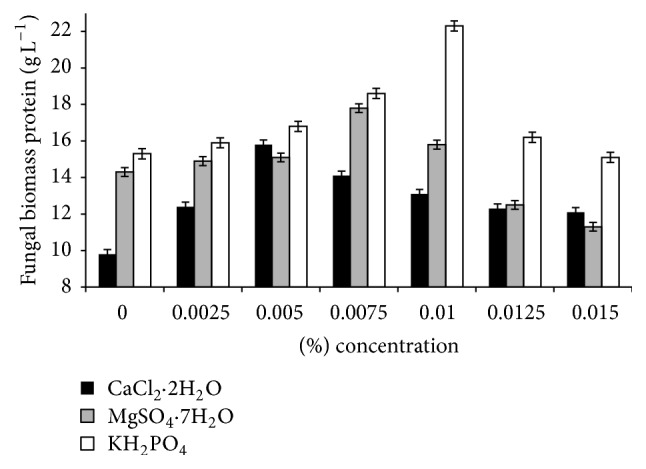
Effect of various levels of ionic concentration on fungal biomass protein production from rice polishings by* T. harzianum*.

Economic production of FBP from* T. harzianum* is required for its production at industrial scale. In order to investigate the possibilities of improving microbial performance, the effect of various ionic concentrations sources was studied. Optimum concentration of the variables was found to be 0.1% CaCl_2_·2H_2_O, 0.15% MgSO_4_·7H_2_O, and 0.2% KH_2_PO_4_ (Figure 5). These results suggested that* T. harzianum* could be a useful strain for FBP production due to its low nutrient requirements. Hence we have formulated a medium with optimized ionic concentrations for the enhanced production of FBP from rice polishings with* T. harzianum*. The predetermined conditions which were optimized to produce FBP during fermentation of rice polishings with* T. harzianum* such as 5% (w/v) rice polishings as a substrate, 0.1% CaCl_2_·2H_2_O, 0.15% MgSO_4_·7H_2_O, and 0.2% KH_2_PO_4_ at 28°C (pH 4.0, 72 h) were used in all succeeding experiments.

### 3.6. Effect of Carbon: Nitrogen Ratio on FBP Production

**Figure 6:**
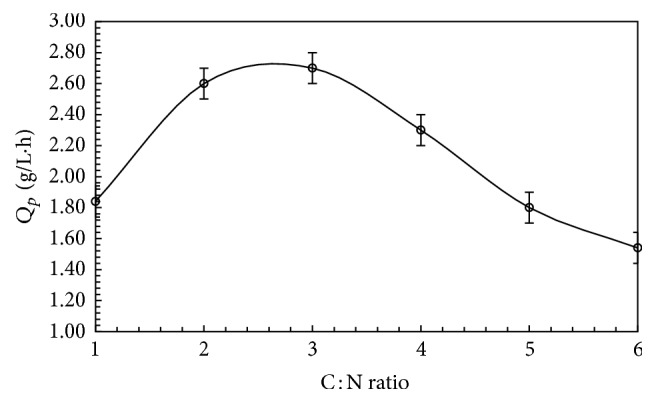
Effect of different C : N ratio FBP protein production by* T. harzianum* grown on rice polishings. 1, 2, 3, 4, 5, and 6 stand for C : N ratios of 30 : 1, 25 : 1, 20 : 1, 15 : 1, 10 : 1, and 5 : 1. Error bars show standard deviation among *n* = 3 readings.

Carbon nitrogen ratio in fermentation process influences fermentation process for production of protein concentrates. To reveal the influence of this ratio on production of biomass protein, it was obtained by increasing concentration of urea in the medium. C : N ratio of 20 : 1 gave significantly higher FBP production (Figure 6). A reasonable amount of C : N ratio is the key to harvest high quality MBP [21]. A C : N ratio of 8 : 10 has been reported for biomass production [22]. The results generally confirmed that urea which is a lost cost fertilizer supports maximum FBP production from* T. harzianum*.

### 3.7. Kinetics and Thermodynamic Parameters of Crude Protein and Cell Mass Production from* T. harzianum*

The results of effect of various substrate concentrations (rice polishings) on kinetic parameters of crude protein and cell mass formation are given in Table 1. It has been shown by analysis of variance (results not shown) that various concentrations of rice polishings possessed significant effect on cell mass production and crude protein as well as utilization of substrate. Effect of various concentrations of rice polishings on product formation kinetic parameters was highly significant on *Q*_CP_ (*P* values > 0.001), *Y*_CP/*X*_ (*P* > 0.0096), and *q*_CP_ (*P* > 0.001) but nonsignificant (*P* > 0.1023) on cell mass yield (*Y*_*X*/*S*_). Among concentrations of substrate, 50 g substrate/L also supported the parameters of higher substrate utilization and this concentration varied significantly higher in supporting *Q*_*s*_, *Q*_*X*_, and *Q*_CP_. Thus 50 g rice polishings per liter were seen to be suitable to support higher crude protein and cell mass productivity and other kinetic parameters. Rice polishing is a cheap fermentation source and is economically more appropriate for fermentation process [17].

Use of cheap and complex sugar substrates, namely, starchy waste materials and other agroindustrial wastes, is common in industrial fermentation but their usage depends on their geographical location and accessibility. Agroindustrial wastes are reported extensively for the production of SCP [23, 24]. Here we showed that rice polishing was much more effective in increased crude protein and cell mass production.

Thermodynamic parameters to produce crude protein from* T. harzianum* at various temperatures are given in Table 2. Thermodynamic parameters were found to be lower when compared to those reported previously [25] but compared auspiciously with those assessed for a number of various whole-cell bioprocesses. The phenomenon responsible for thermal inactivation is lower than that of production of SCP and significantly lower than the previous given values of 160–235 kJ/mol [26, 27]. This suggests that decrease in productivities which are detected at high temperature could be because of reversible inactivation of those enzymes that are involved in rice polishings metabolism.

**Figure 7:**
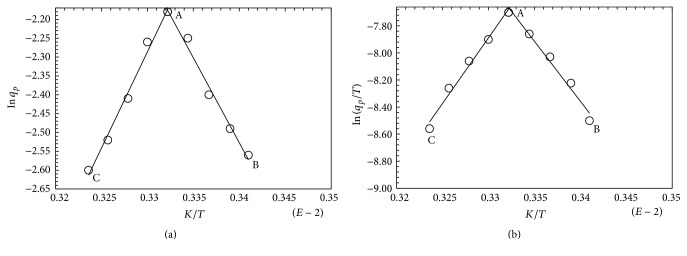
(a) Determination of activation energy for FBP production activation and deactivation network. (b) Determination of enthalpy and entropy for activation of FBP formation and inactivation equilibria of FBP in* T. harzianum* following growth on rice polishings medium under optimized condition. AB is one line and AC is the second line whose slopes have been calculated statistically to determine entropy and enthalpy values.

Activation enthalpy of FBP production was calculated graphically from Figure 7 through the application of Arrhenius approach [18]. The activation enthalpy values of FBP production are given in Table 3. Organism required 37.5 and 42.57 kJ/mol activation energy for production formation and inactivation pathways, respectively. These values were lower than those needed for product formation in the deactivation phase by mesophilic organism [26] and those of thermotolerant* Kluyveromyces marxianus* [28].

### 3.8. Chemical Composition and Nutritive Value of FBP

*T. harzianum* was grown for 72 h in a 75 L fermenter (50 L working volume) under the optimized conditions. After that FBP product obtained was chemically analyzed and its amino acid profile was also estimated. Chemical analysis of final biomass product obtained by* T. harzianum* was determined on dry weight basis. The results are given in Table 4. The true protein of fungal biomass produced by fermentation of* T. harzianum* in a 75 L fermenter was 32.00%. Thus the protein content was adequate for utilization of FBP as fodder. The fungal biomass of* M. indicus* and* R. oryzae* contained 40% and 51%, respectively [29].

The potential of nutritional value of a protein can primarily be determined by its amino acids composition.* T. harzianum* was grown for 72 h in a 75 L fermenter. Amino acid profile of final FBP product was estimated. As evident from Table 5, the FBP produced contained 16 amino acids. This FBP can serve as valuable microbial protein source. Higher percentage of lysine is present in the* T. harzianum* fungal biomass protein that is a limiting amino acid in those of aquatic feeds. Cofeeding the fungal protein with commercially available protein would address the low contents of methionine and phenylalanine in fungal biomass and can decrease the cost of protein ingredient for feeds.

### 3.9. Biological Evaluation of FBP

The experimental broilers gained weight regularly up to 8 weeks and no mortality/abnormality was observed (results not shown). The highest weight gain was recorded in the chicks fed on ration C followed in descending order by those fed on ration B and control ration (A). It indicated that FBP can partially replace soybean meal in the rations of broiler chicks without having any detrimental effects on weight gain of birds (results not shown). The results of the present study are in line with that of Joshi et al. [30] who produced animal feed from apple pomace on large scale by solid state fermentation with five yeasts. Fermented apple pomace mixed with a standard broiler feed (1 : 1 ratio) was comparable to the standard feed.

The lowest feed consumption was recorded on standard ration (A). Maximum feed was consumed by chicks fed on ration (C) in which 60 percent of the soybean protein was replaced with FBP. The results of the present study are in line with that of Ikram-ul-Haq [31] who produced single cell protein from* Brevibacterium flavum* and* Arachniotus* sp. while the substrate used for the microbial protein production was rice polishings. He replaced this microbial protein with that of fish meal.

## 4. Conclusion

FBP produced by this fungus can be used as rich source of protein supplementation in animal feed. Rice polishing (5%, w/v) has shown excellent potential as a substrate for MBP production. Optimum pH, temperature, and time of incubation for FBP production from* T. harzianum* were 4, 28°C, and 72 h, respectively. C : N ratio of 20 : 1 gave significantly higher FBP production. The production of this FBP from rice polishings as a substrate appears to be a technically feasible process for bioconversion of agricultural wastes. From the results, it could be deduced that rice polishing is a good substrate for FBP production to be supplemented in animal feed. The results obtained are considered useful for the production of* T. harzianum* biomass protein on a large scale and can be widely applied to other fungal fermentation.

## Figures and Tables

**Table 1:** Kinetic parameters for crude protein and cell mass production along with substrate utilization by *T. harzianum* in 5-L fermenter under optimized fermentation conditions.

Kinetic parameters	40	50	60	70	*P*
*µ* (h^−1^)	0.15 ± 0.01^b^	0.21 ± 0.01^a^	0.23 ± 0.01^a^	0.09 ± 0.01^c^	0.0001
*Q*_CP_ (g/lh)	0.21 ± 0.01^b,c^	0.24 ± 0.01^a,b^	0.27 ± 0.02^a^	0.19 ± 0.01^c^	0.0001
*Y*_CP/*X*_ (g/g cells)	0.47 ± 0.01^a^	0.49 ± 0.01^a^	0.49 ± 0.01^a^	0.43 ± 0.01^b^	0.0001
*Y*_CP/*S*_ (g/g S)	0.21 ± 0.05^a^	0.23 ± 0.02^a^	0.23 ± 0.05^a^	0.20 ± 0.01^a^	0.698
*q*_CP_ (g/g/h)	0.032 ± 0.004^c^	0.103 ± 0.002^b^	0.113 ± 0.003^a^	0.039 ± 0.002^c^	0.0001
*Q*_*S*_ (g S/lh )	0.88 ± 0.02^c^	1.25 ± 0.02^a^	1.13 ± 0.03^b^	0.56 ± 0.03^d^	0.0001
*Q*_*X*_ (g Cell/l h)	0.39 ± 0.04^a^	0.48 ± 0.05^a^	0.42 ± 0.05^a^	0.39 ± 0.05^a^	0.146
*Y*_*X/S*_ (g cells/g S)	0.45 ± 0.03^a^	0.50 ± 0.03^a^	0.46 ± 0.03^a^	0.43 ± 0.03^a^	0.102

Values are mean ± SD of *n* = 3 experiments.

^a,b,c,d^Means ± SD followed by different superscripts in each row are significantly different at confidence level *P* ≤ 0.05 using Tukey's multiple range test.

**Table 2:** Thermodynamic parameters for reversible activation and irreversible inactivation of SCP production pathway of *T. harzianum*.

Temp. (K)	Temp. (°C)	*q*_*p*_ (g/gh) × 10^−2^	Δ*H*^*∗*^ (kJ mol^−1^)	Δ*G*^*∗*^ (kJ mol^−1^)	Δ*S*^*∗*^ (J mol^−1^ K^−1^)
293	20	8.5 ± 0.44^c,d^	35.06 ± 2^a^	72.52 ± 3.5^b,c^	−127.85 ± 8^a,b^
295	22	9.2 ± 0.45^c^	35.05 ± 2^a^	78.49 ± 3.5^a,b,c^	−147.25 ± 9^b,c^
297	24	9.7 ± 0.45^b,c^	35.045 ± 2^a^	82.76 ± 4^a,b^	−160.66 ± 10^c^
299	26	10.6 ± 0.51^a,b^	35.01 ± 2^a^	74.14 ± 3.5^a,b,c^	−231.2 ± 17^d^
301	28	11.3 ± 0.55^a^	37.0 ± 2.3^a^	67.66 ± 3.5^c^	−101.86 ± 7^a^
303	30	9.4 ± 0.41^b,c^	34.08 ± 2^a^	80.2 ± 4^a,b^	−152.21 ± 10^b,c^
305	32	8.8 ± 0.42^c,d^	34.06 ± 2^a^	80.91 ± 4^a,b^	−153.61 ± 10^b,c^
307	34	7.6 ± 0.43^d,e^	34.05 ± 2^a^	81.83 ± 4^a,b^	−155.64 ± 10^b,c^
309	36	6.6 ± 0.34^e,f^	34.03 ± 2^a^	82.75 ± 4^a,b^	−157.67 ± 10^b,c^
311	38	5.8 ± 0.34^f^	34.02 ± 2^a^	83.63 ± 4^a^	−159.52 ± 10^c^

*P*		0.0001	0.754	0.0001	0.0001

^a,b,c,d,e,f^Values followed by different superscripts in each column differ significantly at confidence level *P* ≤ 0.05 using Tukey's multiple range test.

Δ*G*^*∗*^ (kJ mol^−1^) = −*RT*ln⁡(*q*_*P*_ · *h*)/(*k*_*B*_ · *T*) and Δ*S*^*∗*^ = (Δ*H*^*∗*^ − Δ*G*^*∗*^)/*T*.

Δ*H*^*∗*^, Δ*G*^*∗*^, and Δ*S*^*∗*^ are enthalpy, Gibbs free energy, and entropy of irreversible inactivation of SCP formation pathway, respectively.

**Table 3:** Thermodynamic parameters for activation and deactivation pathways for FBP production.

Attributes	SCP formation	Thermal inactivation	*P*
Activation energy	37.5 ± 2^b^	42.57 ± 1.5^a^	0.025
Activation enthalpy (kJ/mol)	73.2 ± 3^b^	82.47 ± 2^a^	0.011
Activation entropy (J/mol·K)	−16.88 ± 4^a^	−535 ± 14^b^	0.0001

Each value is a mean of three replicates. Values followed by different superscripts in each row differ significantly at confidence level *P* ≤ 0.05 using Tukey's multiple range test.

**Table 4:** Compositional analysis of rice polishings and biomass (% dry weight).

Components	Rice polishings	Biomass obtained from *T. harzianum *in a 75 L fermenter	*P*
Moisture	6.77 ± 0.2^a^	1.50 ± 0.08^b^	0.0001
Crude protein	11.25 ± 0.5^b^	49.50 ± 1.7^a^	0.0001
True protein	1.0 ± 0.1^b^	32.00 ± 1.3^a^	0.0001
Crude fat	11.73 ± 0.6^b^	21.25 ± 0.74^a^	0.0001
Crude fiber	6.25 ± 0.21^b^	19.45 ± 0.78^a^	0.0001
Ash	9.50 ± 0.4^a^	9.62 ± 0.5^a^	0.762
Nitrogen Free extract	54.50 ± 2.7^a^	28.33 ± 1.1^b^	0.0001
Cellulose	10.76 ± 0.43^a^	11.5 ± 0.46^a^	0.112
RNA	ND	0.325 ± 0.02	ND

ND = not determined; values are mean ± SD of *n* = 3 experiments.

^a,b^Means ± SD followed by different superscripts in each row are significantly different at confidence level *P* ≤ 0.05 using Tukey's multiple range test.

**Table 5:** Amino acid profile of biomass by *T. harzianum *in 75 L fermenter.

S. number	Amino acid	Rice polishings (%)	Biomass% produced in a 75 L fermenter by *T. harzianum*	*P*
1	Aspartic acid	0.76 ± 0.04^b^	1.05 ± 0.04^a^	0.0004
2	Threonine	0.43 ± 0.02^b^	0.70 ± 0.03^a^	0.0001
3	Serine	0.51 ± 0.03^b^	0.75 ± 0.04^a^	0.0001
4	Glutamic acid	1.80 ± 0.05^b^	4.76 ± 0.17^a^	0.0001
5	Proline	0.74 ± 0.04^b^	1.85 ± 0.074^a^	0.0001
6	Glycine	0.55 ± 0.03^b^	0.76 ± 0.03^a^	0.0005
7	Alanine	0.62 ± 0.03^b^	0.86 ± 0.03^a^	0.001
8	Valine	0.61 ± 0.03^b^	1.05 ± 0.05^a^	0.0001
9	Methionine	0.00	0.22 ± 0.01	ND
10	Isoleucine	0.37 ± 0.02^b^	0.69 ± 0.024^a^	0.0001
11	Leucine	0.68 ± 0.03^b^	1.18 ± 0.04^a^	0.0001
12	Tyrosine	0.27 ± 0.02^b^	0.51 ± 0.03^a^	0.0002
13	Phenylalanine	0.55 ± 0.025^b^	1.00 ± 0.03^a^	0.0001
14	Lysine	0.57 ± 0.03^b^	21.34 ± 0.75^a^	0.0001
15	Histidine	0.30 ± 0.01^b^	0.73 ± 0.04^a^	0.0001
16	Arginine	0.66 ± 0.04^b^	1.14 ± 0.06^a^	0.0003

Test samples were hydrolyzed with HCl and analyzed using an automated amino acid analyzer.

ND = not determined; values are mean ± SD of *n* = 3 experiments.

^a,b^Means ± SD followed by different superscripts in each row are significantly different at confidence level *P* ≤ 0.05 using Tukey's multiple range test.
